# Keep Calm and Stay Safe: The Relationship between Anxiety and Other Psychological Factors, Media Exposure and Compliance with COVID-19 Regulations

**DOI:** 10.3390/ijerph18062852

**Published:** 2021-03-11

**Authors:** Tomer Mevorach, Jonathan Cohen, Alan Apter

**Affiliations:** 1 Department of Psychological Medicine, Schneider Children’s Medical Center of Israel, Petah Tikva 4920235, Israel; eapter@clalit.org.il; 2 Sackler Faculty of Medicine, Tel Aviv University, Tel Aviv 69978, Israel; 3 Department of Communication, University of Haifa, Mt. Carmel, Haifa 31905, Israel; jcohen@com.haifa.ac.il

**Keywords:** anxiety, COVID-19, compliance, media exposure, uncertainty

## Abstract

The COVID-19 pandemic has led many countries to employ public health regulations to achieve behavioral change and stop the transmission of the virus. The factors influencing compliance with these regulations may differ from “classic” predictors for medical compliance. This study attempted to assess the effect of social communication and psychological factors on intention to comply. A cross-sectional online survey was conducted on healthy adults living in Israel (*n* = 697). The survey assessed the intention to comply with the state COVID-19 regulations and explored possible correlations with demographic and psychosocial factors. Data were collected during May 2020 using a Qualtrics online survey. Data were analyzed to find correlations between anxiety, uncertainty, media exposure and other variables and the level of intention to comply as self-reported. Moderation and mediation effects were studied by an integrative model of influencing factors. We found that media exposure change, trust in responsible agencies and anxiety were positively correlated with compliance, while uncertainty was correlated with noncompliance. The effect of media exposure on compliance had two components. First, media exposure was positively correlated with compliance. On the other hand, media exposure was positively correlated with uncertainty, and uncertainty was negatively correlated with compliance. Interestingly, anxiety, which was positively correlated with media exposure, also moderated the negative correlation between uncertainty and compliance. Our results highlight the important role of uncertainty and anxiety as moderators between media exposure and compliance. To increase public compliance with COVID-19 regulations, efforts should be directed at decreasing uncertainty and anxiety.

## 1. Introduction

The COVID-19 pandemic constitutes a global crisis and presents a serious challenge in attempting to control the spread of the disease. In the absence of medications or vaccines, many countries implemented restrictions on their citizens’ behavior, intended to limit transmission rates. On 26 January 2020, the Israeli Ministry of Health declared the novel coronavirus disease (COVID-19) to be of international importance, thus authorizing the Ministry of Health to take special measures for coping with the pandemic (Israel Ministry of Justice, 2020). Over the following three months, the Ministry of Health issued instructions, first requiring quarantine for anyone entering the country (4 March), then banning public gatherings (10 March), then closing schools and childcare facilities (12 March). On 19 March, the ministry published guidelines for social distancing and rules for sanitation and public lockdown (Israel Ministry of Health, 2020).

The effectiveness of such regulations depends on the public’s level of compliance, which often varies among social groups and locations and over time. Factors influencing compliance have been previously studied. The current situation is highly challenging for the population because regulations change often, at short notice, and involve numerous government agencies and other national agencies that represent opposing interests and have difficulty working together.

A recent study on compliance with COVID-19 regulations demonstrated that proper compensation for lost wages due to the restrictions may elevate rates of compliance [1]. In another recent study, Liu et al. [2] discuss the 3C model as a predictor of adoption or nonadoption of preventive behaviors. The 3Cs are confidence in social institutions; complacency regarding one’s risk of infection and constraints, which relates to levels of self-efficacy; and confidence in one’s ability to engage in the behaviors. Other predictors of nonadherence may include ADHD (attention deficit hyperactivity disorder) symptoms, psychological distress and previous engagement in risk-taking behaviors [3].

The present study aimed to uncover factors influencing people’s intention to comply with regulations. Previous studies found compliance to differ by gender, although findings are controversial regarding which is the more compliant gender [4,5]. Compliance may also differ by ethnicity, as found in some studies [6,7,8] but refuted by the findings of another study [9]. These differences in findings may be explained by Abel et al. [10], who showed that compliance may depend on the levels of trust between the patient and the healthcare provider, who in this case is represented by the government agencies managing the epidemic. Trust in the government seems indeed to be a predictor of compliance with the regulations, as demonstrated in several recent studies [11,12,13,14].

Some mental states may also constitute predictive factors for compliance with regulations, such as depression and anxiety resulting from the social isolation and the uncertainty and fear caused by the pandemic [15,16,17]. Both depression and anxiety have been shown to have negative effects on compliance [18], which may be explained by the cognitive biases co-occurring with anxiety [19], such as interpretation and attention biases [20] and harm-avoidance tendencies [21].

As previously mentioned, uncertainty stemming from the pandemic may lead to increased anxiety [22,23] and an increase in the prevalence and influence of cognitive biases in assessing threat [24]. Research into the effects of uncertainty on the public has found that they are correlated with lower levels of medical compliance [25,26] and lower levels of mental well-being [27]. Uncertainty may lead to less compliance because it is associated with anxiety [28,29], and it may interrupt goal-directed functioning [30] and promote distress [31]. Conversely, Akesson et al. [32] showed that offering accurate and reliable information on the rate of COVID-19 infection had a positive impact on the public’s willingness to comply with regulations. 

The media plays a critical role in publishing regulations, disseminating relevant information and promoting public adherence. According to the protective action decision model (PADM) [33], access to and preferences among information sources, such as media, are important determinants of behavioral responses such as the decision of whether to comply. Dryhurst et al. [34] identified a number of variables correlating with risk perceptions of COVID-19, among them personal experience, knowledge about the pandemic and trust in government and medical professionals. Though media, especially social media, may also be a source of misinformation regarding the pandemic, exposure to reliable news media has been found to contribute to less misinformation, improved risk perception and promotion of increased social distancing [35].

This study attempted to assess the effect of social communication factors such as media exposure, institutional trust and political orientation on intention to comply. The mutual interaction between psychological and communicational factors was a special focus of interest.

## 2. Materials and Methods

### 2.1. Sample

Participants (*n* = 932) were recruited through an Israeli commercial online panel that sent a link to a Qualtrics survey. Inclusion criteria were adults (over the age of 18 years) who were fluent in Hebrew. Within the questionnaire was a short attention check to verify the participants’ ability to attentionally complete the survey. In total, 121 participants failed the attention check and were excluded from the study. Participants who did not complete the survey (*n* = 78) or who completed the survey in less than 4.5 min (*n* = 36) were also excluded from the study. A total of 697 participants were finally included in the study. The study was approved by an institutional Helsinki committee. The survey included an informed consent procedure, and participants were compensated for their participation.

### 2.2. Procedure

The survey took place from 12 to 21 May 2020. The State of Israel was under full lockdown in the month prior to the study. During the survey period, the quarantine restrictions were gradually lifted and the number of new SARS-CoV-2 patients was low. Out-of-home restrictions were lifted while schools, markets, restaurants and public places were closed. The survey compliance questionnaire matched the COVID-19 regulations at that time.

Demographics: Participants were asked to report their age, sex (male/female/other), educational level (graduate school, college graduate, partial college education, high school) and religiosity (secular, religious, ultraorthodox, other). Political position was measured in the three scales of security, economics and social issues.

COVID-19-related information: Participants were asked about their risk factors for severe COVID-19 symptoms as well as high-risk factors in their close relationships. Participants were also questioned about diagnosis of SARS-CoV-2 infection and isolation as a result of exposure to a verified SARS-CoV-2 patient.

Compliance: Intended compliance was assessed by a seven-item questionnaire. Participants rated from 1 to 10 their intention to comply with each of the following COVID-19 regulations in the near future: wearing a mask in public, avoiding gatherings beyond the permitted number, maintaining social distancing, frequent hand washing, measuring temperature before leaving the house for public places, limits on the number of passengers riding together in private cars and limits on the number of people taking an elevator together.

Media exposure: To assess media exposure, participants were asked to report the extent to which their exposure to COVID-19-related news and information had changed during the COVID-19 crisis period. Participants were asked about different media sources such as television, radio, web news sites, social networks and newspapers.

Trust in officials and public institutions: Participants were asked to report their levels of trust in 11 officials and public institutions related to the COVID-19 crisis, using a Likert scale of 1 (no trust at all) to 7 (complete trust). Based on the accepted definition of credibility (Podsakoff, 1990), trust was defined as the belief that decisions made by officials or public institutions are honest, professional and aimed at the benefit of the public. Officials (by name) included the Prime Minister, Minister of Health, Minister of Finance, Director-General of the Ministry of Health and Head of Public Health Services of the Ministry of Health. Public institutions included the government, professional functions in the Health Ministry, professional functions in the Ministry of Finance, hospitals, the news media and the army’s headquarters.

Degree of knowledge: To assess participants’ knowledge about the COVID-19 pandemic, a short knowledge questionnaire was used. Participants were tested for their knowledge about pandemic spreading, common COVID-19 clinical symptoms, means for viral disinfection and understanding of the term “incubation period”. 

Anxiety: Anxiety was measured by the General Anxiety Disorder-7 (GAD-7) scale.

Uncertainty: Uncertainty was measured by agreement degree with five uncertainty-related statements on a 1–5 scale (e.g., “I understand how Israel will deal with COVID-19 pandemic”; “I know how COVID-19 will affect my life in the coming months”). 

Data were collected using Qualtrics.^XM^ (Qualtrics, Provo, UT, USA), and the data analysis was processed with IBM SPSS statistics (IBM Corp, Version 27.0. Armonk, NY, USA). Moderation and mediation analysis used a structural equation computation model (AMOS) and a Hayes’ (2013) [36] process approach utilizing process model 6 (serial mediation).

## 3. Results

Participants’ demographics are presented in Table 1. Test score reliability was high for the compliance, media exposure, trust in officials and public institutions, anxiety and uncertainty (Cronbach’s alpha > 85%) and lower for degree of knowledge (Cronbach’s alpha = 65%). 

The reported intention to comply with the COVID-19 regulations was 7.60 (±1.92). As demonstrated in Table 2, there was a slight variability between the regulations.

The political orientation of the sample was found to be close to the midpoint for security (M = 63.17, SD = 29.07), economics (M = 54.71, SD = 27.04) and social issues (M = 51.78, SD = 29.61). An additional new variable of corona exposure risk was calculated by summing up the four relevant items (M = 0.82, SD = 0.77). Knowledge was also summed up from four questions to one variable (m = 3.71, SD = 0.51). The total media exposure change was positive (M = 3.42, SD = 0.79). Public trust varied across agencies (see Table 3 for descriptive results).

In the psychological part of the study, anxiety was found to be relatively low (m = 1.65, SD = 0.71), and uncertainty about coping with the pandemic was below the midpoint of the scale (m = 2.95, SD = 0.87).

**Figure 1:**
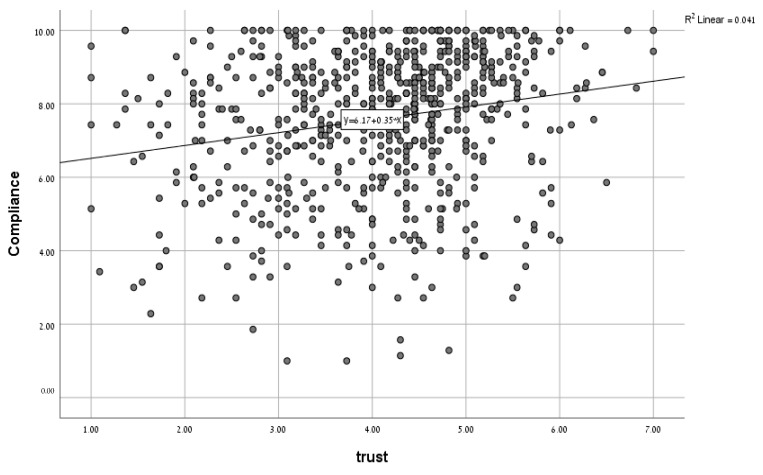

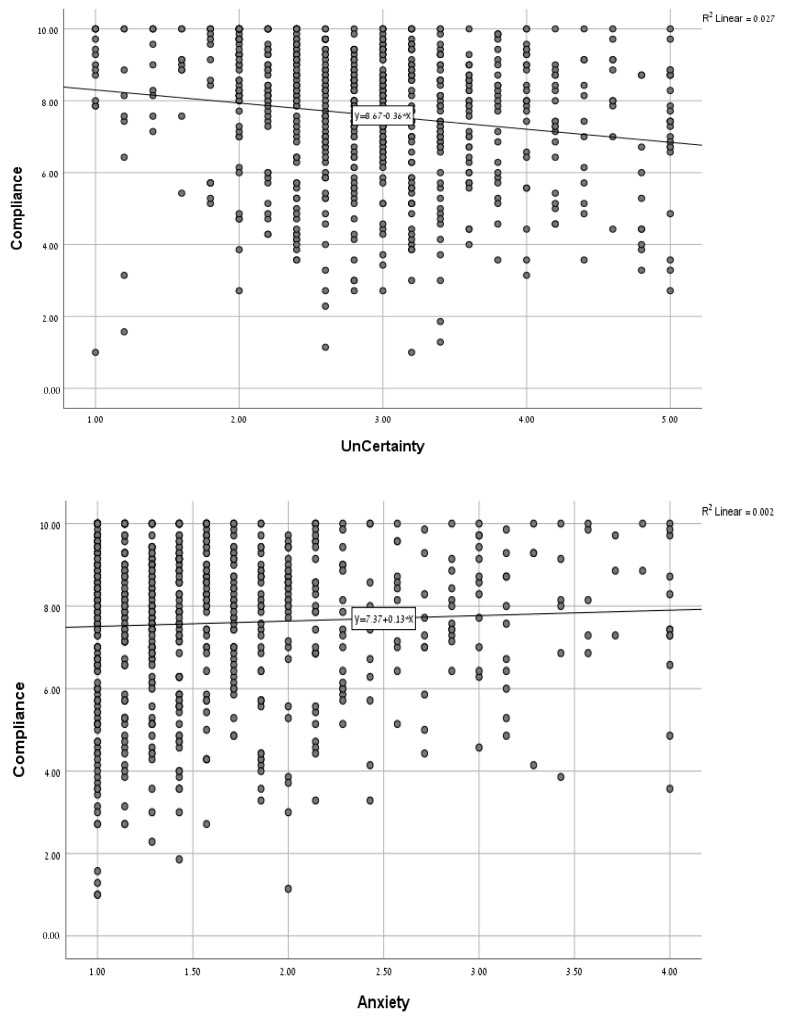
Associations of single factors with the level of intention to comply, continuous variables in Pearson correlation graph.

Simple associations between main possible predictors and intention to comply were tested using the Pearson correlation coefficient as depicted in Figure 1.

Regression analysis was performed to quantify the correlation of each variable with compliance, controlling the effect of other variables, and to find the total prediction rate of all variables together (Table 4). Overall, the variance explained by all factors was rather low (adjusted R square = 0.13), with demographics and media exposure adding significant contributions. Sex was a predictor of compliance, with women reporting more intended compliance than men (b = −0.36, SE = 0.14, *p* < 0.05). Older respondents reported more intent to comply (b = 0.03, SE = 0.01, *p* < 0.01), as did Arabs (b = 0.56, SE = 0.23, *p* < 0.05). None of the political dimensions proved to be a significant predictor. Media exposure change was a positive predictor (b = 0.35, SE = 0.01, *p* < 0.01); however, knowledge about COVID-19 did not prove to be a significant predictor (b = −0.02, SE = 0.14, ns). Trust was in positive correlation with intent to comply (b = 0.28, SE = 0.07, *p* < 0.01), and uncertainty was found to negatively correlate with intent to comply (b = −0.25, SE = 0.09, *p* < 0.05). Anxiety was also positively correlated with intent to comply, but this correlation was only marginally significant (b = 0.20, SE = 0.10, *p* < 0.06). The regression was made again separately on subgroups of the sample, assuming that demographic parameters may confound the power of the regression analysis on the whole samples. The results didn’t change robustly, as can be seen in Table 5.

**Figure 2:**
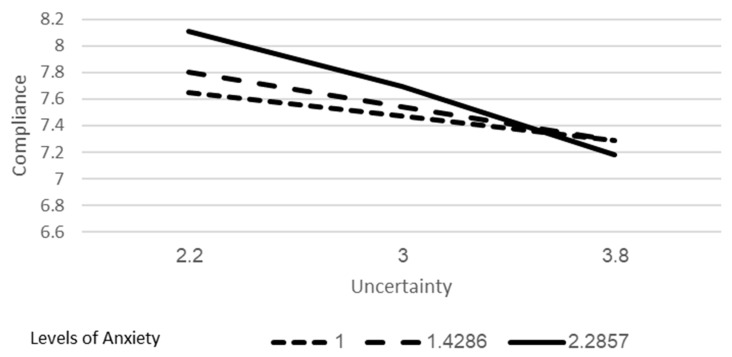
Moderating effect of anxiety on the relationship between uncertainty and compliance.

The mediation model depicted in Figure 2 predicts that compliance would be a direct function of media exposure and that this relationship would also be mediated through anxiety and uncertainty, in turn. In addition, a direct link from anxiety to compliance as well as an interaction between anxiety and uncertainty was modeled based on our expectation that the effects of uncertainty on compliance would be moderated by anxiety. In addition, the interaction term was allowed to correlate with error terms of both its constituent parts. The model fit the data very well (CFI (Comparative fit index) = 0.99, NFI (normed fit index) = 0.99, 1-RMSEA (root-mean-square error of approximation) = 0.92). Process approach analysis found that changes in media exposure indirectly influenced intent to comply through their effect on anxiety and uncertainty. Media exposure positively influenced anxiety (b = 0.10, SE = 0.03, 95% CI [0.04, 0.17]), and anxiety influenced uncertainty (b = 0.12, SE = 0.05, 95% CI [0.03, 0.21]). As expected, uncertainty negatively influenced compliance (−0.35, SE = 0.08, 95% CI [−0.51, −0.18]). The direct effect of media exposure on compliance was positive (b = 0.38, SE = 0.09, 95% CI [0.20, 0.56]), but the total indirect effect was weaker, negative and significant (b = −0.01, SE = 0.01, 95% CI [−0.01, −0.01]) because of the negative effect of uncertainty.

**Figure 3:**
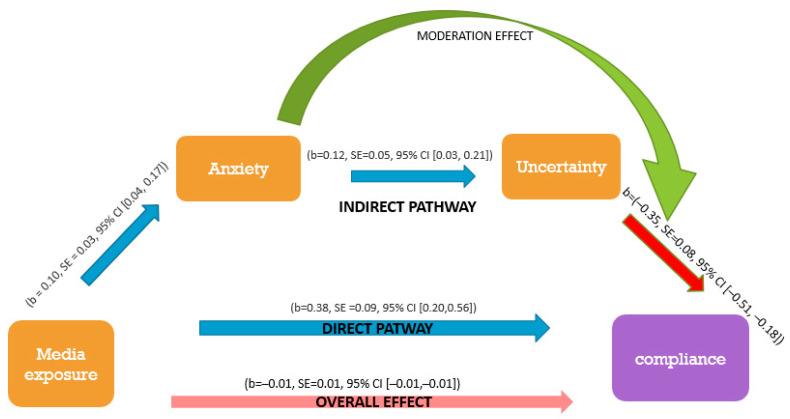
General model of the influence of media exposure change on the intention to comply, including anxiety and uncertainty as mediators. The model fits our data well as described in the body of the text. The moderation effect in the green arrow specified in Figure 2.

As can be seen in Figure 3, using the Johnson–Neyman method [37] to explore the moderation, it was found that at all levels of anxiety, the relationship between uncertainty and compliance is negative and significant. However, whereas at the lowest level of anxiety (1) that relationship is of moderate magnitude (b = −23), at the highest level of stress (4) that relationship is more than 3 times as strong (b = −0.91).

## 4. Discussion

This study examined predictors of intention to comply with government regulations regarding COVID-19 in the Israeli context. The data were collected when the pandemic had already caused immense disruption of social life but seemed to be under control. At this point, the reported intention to comply was very high and quite uniformly so, which could explain why the overall explained variance in our study was not high. Nonetheless, several variables proved to be theoretically consistent and significant predictors of compliance, and interesting interrelationships between the predictors were found. 

The factors that showed significant positive associations included trust, media exposure and anxiety, whereas uncertainty was a significant and negative predictor. Overall, our findings were consistent with previous research on classic medical compliance and adherence [12,18,38], even though the present context of governmental regulations about COVID-19 was quite different. 

Our findings about demographic predictors were partly surprising. Previous research has reported heterogeneous findings regarding gender gaps in compliance [8,39]. In our study, there was a significant gender difference in that women reported more intent to comply than men. As to social minorities, much public interest was raised regarding compliance among the Haredi (ultraorthodox) community, which in this research showed no difference from the general population. Nevertheless, the Arab minority, which was also singled out, reported higher rates of intention to comply. This is especially interesting since trust in leadership is a predictor of compliance [40], and one would suspect Arabs, as a minority, would have less trust in leadership due to underrepresentation in decision-making circles.

Of special interest were the mediation and moderation effects between the different factors. Media exposure was found to have a complex relationship with compliance, mediated and moderated by anxiety and uncertainty. While the direct effect of media exposure on compliance was positive, it also had a positive effect on anxiety and uncertainty, which in turn decreased the intent to comply. In other words, media exposure had both a positive direct effect on compliance and negative indirect effects. In summary, controlling for anxiety and uncertainty, being more attentive to media increased compliance, but it also increased anxiety and uncertainty, which in turn reduced compliance.

This complicated relationship may clarify the role of media in a public health crisis such as the current pandemic. Media are the major tool for the dissemination of information and knowledge about the crisis [34] and thus are a key factor in achieving a good level of compliance. According to media system dependency theory [41], the public’s dependence on media for information increases during times of crisis, and alternative sources of information (e.g., interpersonal) cannot supply reliable guidance and certainty. Garfin et al. [42] suggest from their review of the research on the media’s role in previous public health crises that, “During an ongoing threat from a novel disease outbreak, timely updates from trusted sources about the relative risk of contracting the novel disease versus a more common one are critical. Without them, public fears may escalate, fuel rumors, and provoke stress responses.” (p. 356). This highlights both the importance of effective information dissemination through media and the necessity for information sources to be trusted. Unlike some previous cases cited by Garfin et al. [42] such as Ebola or the Boston Marathon bombing, in the case of COVID-19, the crisis was ongoing and relevant to many, and there were actions that the public was expected to take, thus making the role of media exposure all the more crucial.

Our finding highlights other aspects of the media’s role, suggesting that information about preventive regulations is not neutral information, and by definition such information is colored by an emotional tone of threat and danger, especially in such a major international crisis [43]. Thus, while media exposure can provide crucial information that leads to compliance, it seems to also increase uncertainty, which has a negative effect on compliance. 

From a public policy perspective, these findings emphasize the importance of ensuring that media provide clear information and reflect transparent decision-making, thus increasing levels of trust and reducing uncertainty by letting citizens understand the regulation’s reasons and goals [44]. Media exposure that leads to a moderate level of anxiety was found to actually contribute to compliance, especially if it is not coupled with uncertainty.

Uncertainty was found to be a negative predictor of compliance, but this association was influenced by levels of anxiety. As can be seen in Figure 2, respondents who experienced lower levels of anxiety were affected less by uncertainty than those who experienced high levels of anxiety. Another way of looking at these results is that uncertainty is not a serious problem for compliance for people who are not anxious. When information creates anxiety, uncertainty hinders compliance to a much greater extent. 

The latter finding contributes to our understanding of the mechanism associating anxiety and compliance. In our regression analysis, anxiety was a positive predictor of intent to comply, suggesting that worrying about the pandemic led to better compliance. As mentioned in the introduction, previous studies found mixed results regarding this association [18,24]. Our moderation model suggests that this relationship may vary, depending on the level of uncertainty. A possible explanation is that adapting preventive behaviors is used to obtain a sense of control and help people cope with anxiety. This tool is only helpful when feeling relatively certain about the reasons and causality of behaviors and events, in other words, feeling certainty. On the contrary, uncertainty interrupts this coping mechanism, because action does not seem to provide an assurance of safety and relief from the sense of danger.

## 5. Conclusions

Several factors were found to be correlated with the level of intention to comply with COVID-19 regulations and can be identified as possible predictors for more general social compliance. These include trust in professional and government elites, media exposure and anxiety levels. In contrast, uncertainty was found to be a negative predictor, suggesting that it reduces compliance.

More nuanced analysis demonstrated the interplay of psychological factors in the processing of public health information. Specifically, our data suggested that anxiety and uncertainty have mediating and moderating roles in the relationship between media exposure and compliance. Figure 1 provides a general model of these relationships, showing first that uncertainty and anxiety both mediate the effect of media exposure on compliance. These mediating roles remind us that health information’s impact on audiences is neither direct nor simple and that information provided via mass media may not always have the intended effects. Rather, public health information impacts psychological states, such as uncertainty and anxiety, and these mediate the influence of information and direction provided by elites on willingness to comply. If information is provided in such a way as to create great uncertainty, it may decrease rather than increase the willingness to comply.

A second implication of this study comes from our finding that the association between uncertainty and compliance was moderated by anxiety, as shown in Figure 2. This moderation indicates that in states of high-level anxiety, the capability to overcome uncertainty and continue functioning according to social expectations is interrupted. This is theoretically consistent with what is known regarding the cognitive biases in anxious subjects [20,21,45], but the implication on public behaviors is one of the study’s contributions. Specifically, these findings emphasize the importance, in times of crisis, that information be disseminated via mass media in a manner that balances providing information that impresses the gravity of the situation upon the public and considering the uncertainty and emotional stress felt by the public because of the media during the pandemic. It also emphasizes the importance of accuracy and adequacy of the messages to special populations such as people with mental health problems, who might respond differently from the general population.

### Limitations and Future Research 

Our study has several limitations. Firstly, all the measures came from self-reported questionnaires, and the actual compliance may be different from the intended one. It is not completely clear what the relationship is between predictors and factors influencing reported intent to comply and those affecting actual compliance. Secondly, this study examined factors predicting compliance from various domains, including psychological, social and others. Thus, many confounders may conceal the findings, but the multidisciplinary characteristic is also an important strength. As for the factor of knowledge, it may depend on interpretations, previous experience and levels of education. Further studies are needed to clarify the mechanisms described above, including longitudinal follow-up on a larger number of participants to establish the interrelations between the different factors. Finally, the current data provide a snapshot of the general population, but there is importance in looking at specific populations, such as people with mental health conditions, who may differ significantly in how they respond to media coverage in times of crisis and may require special attention.

## Figures and Tables

**Table 1:** Participants’ demographics.

Characteristic	Participants
Age, mean (± SD), y	38 (14)
Gender, percentage	
Men	44.6
Women	55.4
Ethnicity, percentage	
Jewish population	84.2
Arab population	15.8
Religiosity, percentage	
Secular	48.5
Religious/traditional	31.7
Ultraorthodox	15.8
Education, percentage	
High school graduates	35.2
Undergraduate degree	29.7
Some college education	19.4

**Table 2:** Intention to comply with COVID-19 regulations.

Regulation	Mean Compliance (±SD)	SD
Wearing a mask in public	8.50 ± 2.10	2.10
Avoiding gatherings beyond the permitted number	7.45	2.44
Maintaining social distancing	7.39	2.57
Frequent hand washing	8.72	1.97
Measuring temperature before leaving the house for public places	5.75	3.33
Limits on number of passengers riding together in private cars	7.28	2.99
Limits on number of people taking an elevator together	8.04	2.56
Overall	7.60	1.92

**Table 3:** Means and standard deviations for trust in various state agencies (1–7).

	M	SD
Prime Minister	3.99	2.0
Government	3.53	1.58
Minister of Health	2.94	1.87
Finance Minister	3.36	1.58
Health Ministry professionals	4.66	1.47
Finance Ministry professionals	3.69	1.47
Hospitals	5.13	1.35
Health Ministry Director	4.58	1.67
Director of Public Health	4.43	1.56
News media	3.33	1.57
Army’s home front command	5.22	1.51

**Table 4:** Regression analysis of the probable predictors separately, controlling the effect of other factors (R^2^ = 0.151, adjusted R^2^ = 0.121, F change = 2.042).

Variable (Predictor)	Unstandardized Coefficients		Standardized Coefficients	*t*	Sig.
	B	Std. Error	Beta		
Sex	−0.356	0.144	−0.092	−2.479	0.013
Age	0.029	0.005	0.213	5.441	0.000
Education	0.056	0.067	0.032	0.831	0.406
Ethnicity	0.553	0.228	0.104	2.423	0.016
Political orientation: security	−0.001	0.004	−0.014	−0.237	0.812
Political orientation: economics	0.003	0.004	0.041	0.690	0.491
Political orientation: social issues	0.005	0.004	0.080	1.296	0.195
Corona exposure risk	−0.183	0.093	−0.073	−1.972	0.049
Knowledge	−0.024	0.138	−0.007	−0.177	0.860
Media exposure	0.349	0.090	0.142	3.865	0.000
Uncertainty	−0.250	0.085	−0.112	−2.955	0.003
Anxiety	0.197	0.104	0.072	1.894	0.059
Trust	0.280	0.073	0.162	3.823	0.000

**Table 5:** Differences of the B values (with SDs) between subgroups of the sample, divided according to basic demographic parameters.

	Gender		Age		Education	
	Female	Male	<35	>34	Some College or Lower	Finished College or Higher
Uncertainty	−0.25 (0.121)	−0.33 (0.136)	−0.23 (0.122)	−0.33 (0.119)	−0.13 (0.115)	−0.44 (0.126)
Anxiety	0.18 (0.134)	0.23 (0.167)	0.03 (0.154)	0.32 (0.144)	0.17 (0.137)	0.31 (0.200)
Trust	0.27 (0.097)	0.25 (0.11)	0.16 (0.104)	0.36 (0.106)	0.23 (0.094)	0.025 (0.007)

## Data Availability

Data is available in request by e-mail from the corresponding author.
